# Healthcare professionals’ conceptualizations of palliative care and readiness for early integration: a cross‑sectional mixed‑methods survey in Finland

**DOI:** 10.1186/s12904-026-02194-x

**Published:** 2026-06-16

**Authors:** Ebba Åström, Ella Saaranen, Heidi Andersén, Mikael Johansson, Nelli-Sofia Nåhls

**Affiliations:** 1 Department of Diagnostics and Intervention, Oncology, Umeå University, Umeå, Sweden; 2 Vaasa Oncology Clinic, Wellbeing Services of Ostrobothnia, Vaasa, Finland; 3 Department of Clinical Oncology, Turku University, Turku, Finland

**Keywords:** Palliative care, Early integration, Health professionals, Knowledge and attitudes, Oncology, Early integrated palliative care

## Abstract

**Background:**

Early integration of palliative care for patients with advanced cancer is recommended by major organizations and guidelines, yet palliative care is often still perceived as end-of-life care or as incompatible with active oncological treatment. Such misconceptions may delay timely integration.

**Methods:**

We conducted a cross-sectional mixed-methods survey among healthcare professionals in oncology services in Ostrobothnia, Finland. The survey included 15 Likert-scale statements (1–5) addressing perceived knowledge, values and role beliefs, and perceived capacity and support, plus one free-text question: “What is palliative care?”. Likert responses were summarized descriptively and compared across demographic groups using non-parametric tests. Free-text responses were coded using a predefined framework of 25 themes derived from the WHO definition of palliative care and Finnish national quality recommendations, generating a binary concept-coverage score (0–25). Responses were also classified for an end-of-life/non-concurrent conceptualization (“non-concurrent framing”). Group differences in concept-coverage scores were tested using Mann–Whitney U.

**Results:**

Ninety-six professionals completed the survey (response rate 88.1%); 95 responded to the free-text item. While participants strongly endorsed statements about palliative care benefits and values, endorsement was lower for perceived competence and lowest for resources and support. Nearly one-third of participants expressed a non-concurrent/end-of-life framing of palliative care in their free-text definition. Concept-coverage scores were significantly lower among participants with non-concurrent framing compared with those without, particularly framework categories related to practical factors and system-level drivers.

**Conclusion:**

The open-ended responses indicated variability in the conceptualization of palliative care, including descriptions consistent with an end-of-life or non-concurrent view. This pattern identifies a potential target for education and implementation aligned with national guidance and quality recommendations.

**Supplementary Information:**

The online version contains supplementary material available at 10.1186/s12904-026-02194-x.

## Introduction

Palliative care is an integral part of comprehensive healthcare. The World Health Organization (WHO) defines palliative care as an approach that improves the quality of life for patients and their families facing life-threatening diseases [1]. There is a growing consensus of integrating palliative care early among patients with advanced cancer [2–4]. Early integration has been described as initiating palliative care within 8 weeks of diagnosis, while others consider palliative care as early if it is received during the last three months of life [3, 5, 6]. Early integration has been linked to better symptom control, greater patient satisfaction, and higher levels of QoL [7–10]. Among caregivers, the benefits of early palliative care have included less depression and distress [11, 12].

Despite its benefits, early integration of palliative care faces challenges. A common misconception among both healthcare personnel and patients is that palliative care is non-concurrent with oncological treatment, e.g., chemotherapy, or limited to end-of-life care [13–16]. This indicates a gap between the WHO definition and practical understanding and implementation of palliative care [17–19]. Zimmerman et al. compared public perceived knowledge of palliative care with actual knowledge based on the WHO definition, which was grouped into eight components [20]. Even among respondents reporting high knowledge, only 46% correctly identified components from the WHO definition, e.g., early integration and a holistic approach.

Magalhães et al. conducted a systematic review on healthcare professionals’ perceptions of palliative care in several countries worldwide, proposing that a major barrier in Europe was a confusion about the notion of palliative care [21]. The review included a study from Sweden and Norway, but none from Finland. The review mainly summarized perceptions and reported knowledge but did not evaluate accuracy against external standard definitions. In a study by McMillan et al., 88% of 73 physicians in South Africa recognized that palliative care is not equal to hospice care, but only half of the participants preferred palliative care alongside active cancer treatment [22]. This implies that even when clinicians know that palliative care differs from hospice care, they may still not endorse early or concurrent integration with oncological treatment.

There is a scarcity of research on Finnish healthcare professionals’ perception of palliative care. The level of integration, development, and delivery of palliative care varies between countries, limiting the generalizability of studies [23].

We conducted a cross-sectional mixed-methods survey to examine Finnish healthcare professionals’ perceptions and conceptualization of palliative care, combining structured Likert-scale items with a free-text definition.

Specifically, we aimed to [1] describe endorsement of statements addressing perceived knowledge, values and role beliefs, and perceived readiness—including skills, resources, and support—for delivering palliative care; and [2] assess the extent to which free-text definitions reflect guideline-consistent scope and timing of palliative care, including concurrent integration with active oncological treatment.

## Materials and methods

### Study design and setting

We conducted a cross-sectional mixed-methods survey in oncology services in Ostrobothnia, Finland, including the outpatient oncology clinic, oncology ward, and oncology radiation center at Vaasa Central Hospital. Data were collected between June and November 2025.

### Participants recruitment

Eligible participants were healthcare professionals working in the participating oncology units, including physicians, registered nurses (oncology and radiation), and practical nurses. Healthcare professionals aged ≥ 18 years who work with patients with advanced cancer were eligible for inclusion, while those under 18 years of age or not working with patients with advanced cancer were excluded. Some of the participants were approached in connection with a local palliative care education program offered in April, September, and November 2025 and were invited to complete the survey once. Surveys were available electronically and on paper. Written informed consent was obtained prior to participation.

### Survey instrument

The survey was developed at the Vaasa Oncology Clinic in spring 2025 to match the study objectives. Item development was informed by (1) an initial set of anonymous open-ended responses on palliative care (*n* = 155) collected prior to the present study and used exclusively for formative purposes during survey development. These were inductively coded and prioritized using a Delphi approach, and (2) an expert focus group panel (*n* = 14) that identified and ranked additional themes based on literature and consensus to support content validity. The final instrument comprised 15 statements rated on a 5-point Likert scale (1 = strongly disagree to 5 = strongly agree) and one open-ended question: “What is palliative care?”. Demographic variables included preferred language (Finnish/Swedish/English), gender, age group, and highest level of education. The instrument and its translated versions were tested in focus groups as part of the development process.

### Survey translation and administration

The survey was produced in Finnish, Swedish, and English. Back-to-back translation was performed and checked by medical translators to support linguistic and conceptual equivalence. Participants completed the survey via Research Electronic Capture (REDCap, Vanderbilt University, TN) [24] or paper form in their preferred language. The preferred language was based on the language of the consent form.

### Mixed-methods approach and integration

We used a convergent mixed-methods design: quantitative (Likert statements) and qualitative (free-text definitions) data were collected during the same survey period, analyzed separately, and integrated during interpretation by comparing endorsement patterns with concept-coverage results to identify discordance between normative endorsement and conceptual scope. We combined structured endorsement items with a free‑text definition to detect discrepancies between normative agreement and conceptual understanding that may be obscured by ceiling effects in Likert responses. Integration was operationalized using concept-coverage scores reflecting alignment with 25 guideline-derived components and a binary indicator of non-concurrent framing, as detailed in Methods. Quantitative and qualitative components were given equal priority, and integration focused on identifying and interpreting discordance between endorsement patterns and concept coverage. Quotes are attributed using anonymized identifiers (HS‑HPxx); identifiers do not correspond to staff rosters or data‑collection order.

### Quantitative outcomes

Likert statements were treated as ordinal outcomes. Responses were summarized using medians and interquartile ranges (IQR). For visualization, means were additionally reported in selected figures. Group differences across demographic categories (language, gender, education, and age group) were examined using the Kruskal–Wallis’s test. For the statement “Palliative care should only begin during the last weeks of life,” reverse coding was applied so that higher values consistently represented more favorable alignment with early/concurrent palliative care. A two-sided p-value < 0.05 was considered statistically significant.

### Qualitative framework and coding

Free-text responses were analyzed using a framework-based, deductive content approach. A conceptual framework of 25 themes was derived from the World Health Organization definition of palliative care and the Finnish Institute for Health and Welfare (THL) national quality recommendations, grouped into five categories: goals, domains, factors, system drivers, and continuity markers. Each response was coded for presence/absence of each theme (binary: 1 = present, 0 = absent), yielding a total concept-coverage score ranging from 0 to 25, plus category-specific scores. The five categories correspond to the following five higher-order thematic domains: goals of palliative care, core meaning of palliative care, practical enactment in clinical care, system-level and contextual conditions, scope and timing across the illness trajectory. More detailed description is available in Supplementary Fig. 1 and Supplementary Table 3.

### Inductively identified non-concurrent framing

During initial inductive review, some responses framed palliative care as end-of-life care and/or as non-concurrent with active oncological treatment. This framing was operationalized as a binary variable (“non-concurrent framing”) and applied during framework coding. Participants were then compared as “non-concurrent framing” versus “no non-concurrent framing” groups.

### Reliability and consensus

Two researchers independently coded all free-text responses. Discrepancies were resolved through discussion to consensus. Interrater agreement for the finalized coding framework was high (Cohen’s kappa 0.87–1.00).

### Statistical analysis

Analyses were performed using IBM SPSS Statistics (version 28). Associations between Likert items and demographic variables were tested using Kruskal–Wallis. Differences in total and category-specific concept-coverage scores between non-concurrent framing groups were tested using Mann–Whitney U. Educational level was dichotomized into university versus lower education (primary/high school/vocational/University of Applied Sciences), and age was dichotomized into 18–39 versus ≥ 40 years to support group comparisons. Given multiple comparisons, subgroup analyses were considered exploratory; we emphasize effect sizes and pattern consistency over isolated p-values.

### Ethics, consent, and data protection

The study was conducted in accordance with the Declaration of Helsinki and Finnish National Board on Research Integrity (TENK) guidelines. Ethical approval was obtained from the University of Turku (TY/1220/06.01.01/2024). All participants provided written informed consent. Data were collected and managed using REDCap hosted on secure University of Turku servers and handled in compliance with General Data Protection Regulation (GDPR). No personal identifiers were collected; survey responses were anonymized and stored on password-protected servers.

### Use of AI-assisted tools

AI-assisted tools were used for limited, non-substantive purposes. Microsoft Copilot in Excel was used to support calculation of Cohen’s kappa. ChatGPT was used to assist with shortening and editing text, Grammarly was used for language editing, and Perplexity.ai was used to support the literature search process. All outputs were reviewed and verified by the authors.

## Results

### Subject characteristics

Of 108 participants invited, 96 completed the survey, yielding a response rate of 88.1%. Among these, 68 (70.8%) were completed online, and 28 (29.2%) on paper. Among non-responders, 75% were female and 50% over 40 years. E-mail reminders were sent to non-responders.

Of the participants, 87 (90.6%) were female, and most were over 40 years (Table 1). 48 (50%) had an educational level of University of Applied Sciences, whereas 30 (31.3%) had a university degree. Among the participants, 53 (55.2%) were Finnish-speaking and 43 (44.8%) Swedish-speaking.

**Table 1:** Study demographics

Variable	*N* = 96	(%)
Language		
Finnish speaking	53	55.2
Swedish speaking	43	44.8
English speaking	0	0
Age group (years)		
18–29	11	11.5
30–39	23	24.0
40–49	24	25.0
50–59	31	32.3
60+	7	7.3
Gender		
Female	87	90.6
Male	9	9.4
Other / I prefer not to answer	0	0
Highest level of education		
Primary education (primary school, high school, vocational school)	18	18.7
University of Applied Sciences	48	50
University	30	31.3

### Likert-scale survey means

Healthcare personnel’s agreement to Likert-scale statements is shown in Table 2. Healthcare personnel strongly agreed that palliative care significantly improves patients’ quality of life (mean: 4.9, SD ± 0.3) and strongly disagreed that it should be initiated during the last weeks of life (mean: 4.9, SD ± 0.5). They strongly agreed that early palliative care initiation improves patients’ quality of life (mean 4.8, SD ± 0.5), and that dying patients should receive honest answers about their condition (mean 4.8, SD ± 0.4). There was strong agreement on the importance of seamless collaboration between professional groups (mean 4.8, SD ± 0.5). Active participation of family members in care discussions was strongly supported (mean 4.8, SD ± 0.5).

**Table 2:** Mean scores, standard deviation (SD), median, and IQR for palliative care survey statements

Statement	*N* (%)	Mean (SD)	Median (IQR)
1. Palliative care can be initiated at the same time as curative care.	96 (100)	3.7 (1.3)	4.0 (2.0)
2. Early initiation of palliative care improves the patient’s quality of life.	96 (100)	4.8 (0.5)	5.0 (0.0)
3. Effective pain relief does not automatically result in drug dependency.	95 (99)	4.6 (0.7)	5.0 (1.0)
4. Palliative care can prolong a patient’s life	96 (100)	4.4 (0.8)	5.0 (1.0)
5. Palliative care often includes psychosocial and spiritual support	96 (100)	4.8 (0.4)	5.0 (0.0)
6. Palliative care should only begin during the last weeks of life. (Reverse coded)	96 (100)	4.9 (0.5)	5.0 (0.0)
7. Healthcare professionals can help patients prepare for death.	96 (100)	4.5 (0.7)	5.0 (1.0)
8. The active participation of family members in care discussions is an important part of palliative care.	96 (100)	4.8 (0.5)	5.0 (0.0)
9. Dying patients should receive honest answers about their condition.	95 (99)	4.8 (0.4)	5.0 (0.0)
10. I believe that palliative care significantly improves patients’ quality of life	95 (99)	4.9 (0.3)	5.0 (0.0)
11. Spiritual or existential support is as important as physical care in palliative care	96 (100)	4.6 (0.6)	5.0 (1.0)
12. Seamless collaboration between different professional groups is crucial for high-quality palliative care	96 (100)	4.8 (0.5)	5.0 (0.0)
13. I feel that I have sufficient skills to provide high-quality palliative care to patients.	96 (100)	3.8 (1.0)	4.0 (1.0)
14. I have sufficient resources and support to work effectively in palliative care.	96 (100)	3.5 (0.9)	4.0 (1.0)
15. I feel that I receive adequate emotional support when working in a palliative care team	95 (99)	3.7 (0.9)	4.0 (1.0)

**Fig. 1:**
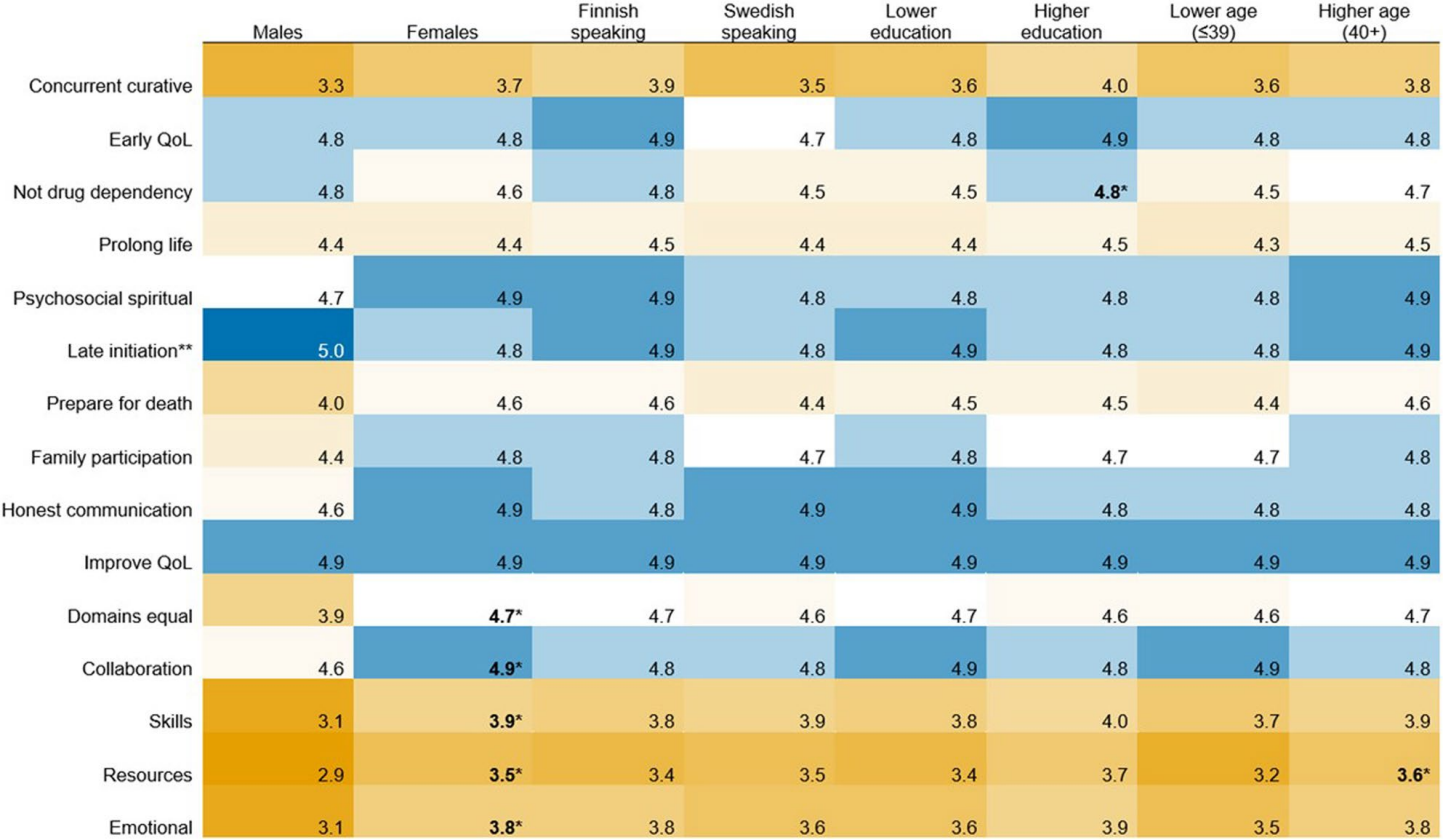
Heatmap of survey statement means by subject characteristics. Possible mean values ranged 1-5; a higher value signified more agreement to survey statement. Blue color indicates more agreement; orange indicates less agreement. Darker color signifies more extreme values. QoL = Quality of Life. * Signifies *p* < 0.05 using Kruskal-Wallis’s test. ** Signifies reverse coded values, e.g. 1 to 5 and 2 to 4, to make all scales aligned so that higher values indicated more favorable resultsPossible mean values ranged 1-5; a higher value signified more agreement to survey statement. Blue color indicates more agreement; orange indicates less agreement. Darker color signifies more extreme values. QoL = Quality of Life. * Signifies *p* < 0.05 using Kruskal-Wallis’s test. ** Signifies reverse coded values, e.g. 1 to 5 and 2 to 4, to make all scales aligned so that higher values indicated more favorable results

Healthcare personnel were neutral or somewhat agreed that palliative care can be initiated at the same time as curative care (mean: 3.7, SD ± 1.3). Similar results were found for readiness statements on having sufficient skills to provide high-quality palliative care to patients (mean 3.8, SD ± 1.0) and feeling that they receive adequate emotional support when working in palliative care (mean: 3.7, SD ± 0.9). Out of all 15 statements, there was the least agreement to have sufficient resources and support to work effectively in palliative care (mean: 3.5, SD ± 0.9). The agreement to survey statements is shown in Fig. 1.

### Likert-scale association to study demographics

There was a significant difference between higher-educated and lower-educated to the statement that pain relief does not automatically result in drug dependency (Fig. 1 and Supplementary Table 1). Both educational groups tended to strongly agree, but healthcare personnel educated at a university had a higher mean value of 4.8 (SD ± 0.5) versus 4.5 (SD ± 0.8), statistically significant when comparing medians and IQR, *p* = 0.035.

Analyzing readiness, a higher age was significantly associated (*p* = 0.045) with more agreement to the statement of having sufficient resources and support to work effectively in palliative care (3.6 SD ± 0.9, vs. 3.2 SD ± 0.9, higher age group vs. lower age group (Fig. 1 and Supplementary Table 1).

### Inductive qualitative thematic analysis of open-ended responses

95 participants responded to the open-ended question, response rate 88.0%. Response length varied, median 21 words (IQR 12–39). Palliative care was recognized among healthcare personnel as a holistic approach addressing multidimensional needs and symptoms as described in the WHO definition of palliative care. The aim of care was described as relief of suffering and improving quality of life, ensuring as good a life as possible. According to healthcare professionals, promoting a normal life for the patient includes support and preventing loss of functional capacity through medical treatments and procedures.

“*The patient is recognized holistically: physical*,* social*,* psychological*,* and spiritual needs are considered.*” (HS-HP01).

*“Symptom-focused care that relieves suffering and aims for the best possible quality of life.”* (HS-HP02).

“*Decreasing tumor size for maintaining*,* e.g*., *stool movement facilitation in colon cancer*, *preventing worse symptoms such as preventing the loss of ability to walk.”* (HS-HP03).

Palliative care was thought to be provided for all patients with an incurable illness, not limited to cancer patients, but including other diseases. However, a limited definition of palliative care being non-concurrent with oncological treatment, initiated only after treatment has ended, or that palliative care is equal to end-of-life care, was described.

“*Good*,* comprehensive care without aggressive oncological treatments”* (HS-HP04).

*“End-of-life care.”* (HS-HP05).

“*We often only think of cancer patients*,* but there are many other illnesses that have palliative care plans. Dementia*,* among many other neurological disorders*,* can be mentioned as examples.*” (HS-HP06).

Healthcare personnel described clear communication as a part of a patient-centered approach. A patient-centered approach included hearing the patients’ perspectives, views, and wishes of care, thus enabling shared decision-making. The need for an advance care plan (ACP) was described, in which, e.g., the limitations and goals of the patient’s care are addressed.

*“Seeing and hearing the person as a whole*,* and respecting individuality as well as one’s own values and convictions.”* (HS-HP07).

*“An advance care plan is important*,* so that everyone (patient*,* relatives*,* healthcare personnel) is on the same page regarding care and plans.”* (HS-HP08).

A multidisciplinary approach, including other professions alongside nurses and physicians, was recognized as important to meet all the patient’s needs comprehensively. This approach included involving and caring for family members, e.g., through providing psychosocial support and bereavement support.

*“… palliative care requires close and seamless collaboration between different operators*,* as successful care is built on a multidisciplinary network.”* (HS-HP09).

*“Considering the patient’s whole family and supporting concretely by arranging*, e.g.,* counseling or preparations for spiritual needs.”* (HS-HP 10).

Early integration of palliative care and its continuation throughout the course of illness were discussed. Some recognized the need for continuous assessment of needs, e.g., by using patient-reported outcomes. Healthcare professionals implied that palliative care should be initiated at the “right time”, but few specified what the “right time” signifies.

“*The patient should be admitted to palliative care well in advance*,* already alongside disease-slowing treatment …”* (HS-HP11).

*” Continuation of care is highlighted. It’s clear for the patient who has responsibility of care and how to contact them.”* (HS-HP12).

*“Assessment of symptoms and support needs at every clinical appointment*,* …”* (P HS-HP13).

Knowledge of palliative care among patients and personnel, including competence, was addressed as part of palliative care delivery. Structural and cultural system factors, e.g., policies, laws, and weaknesses in the care system that influence palliative care delivery, were recognized.

*“Palliative care should be offered to all patients despite of place of residence or age. It’s a human right*,* and the right and availability for it should be written in the law.*” (HS-HP14).

*” Palliative care plays a significant role in healthcare*,* but from a professional’s perspective*,* its importance too often goes unrecognized. Palliative care should be integrated into the care pathways of medical specialties more deeply*,* and collaboration between specialties should be broader.”* (HS-HP11).

Analyzing attitudes and sentiments, providing palliative care was seen as valuable and dignifying, particularly when giving care at the end of life. However, some questioning perspectives were also evident, where the use of resources and provision of treatment late in the disease course were discussed. In some healthcare professionals’ opinions, patients were treated for too long.

*“Good palliative care is of paramount importance … Palliative care is*,* in my opinion*,* the finest medicine that there is.”* (HS-HP07).

*“Sometimes I can question the use of some palliative radiation therapies*,* when the patient comes here and*, e.g.,* gets 1 treatment of 5 planned. The patient can even die before the last radiation dose is given. I understand that sometimes it’s difficult to anticipate the development. But sometimes it feels a bit silly that we should bother the patient when they could have been at peace.”* (HS-HP16).

*“Too often patients are treated by us for too long*,* and the patient more or less dies with an intravenous drip in their hand.”* (HS-HP17).

### Deductive binary coding results of themes in open-ended responses

**Fig. 2:**
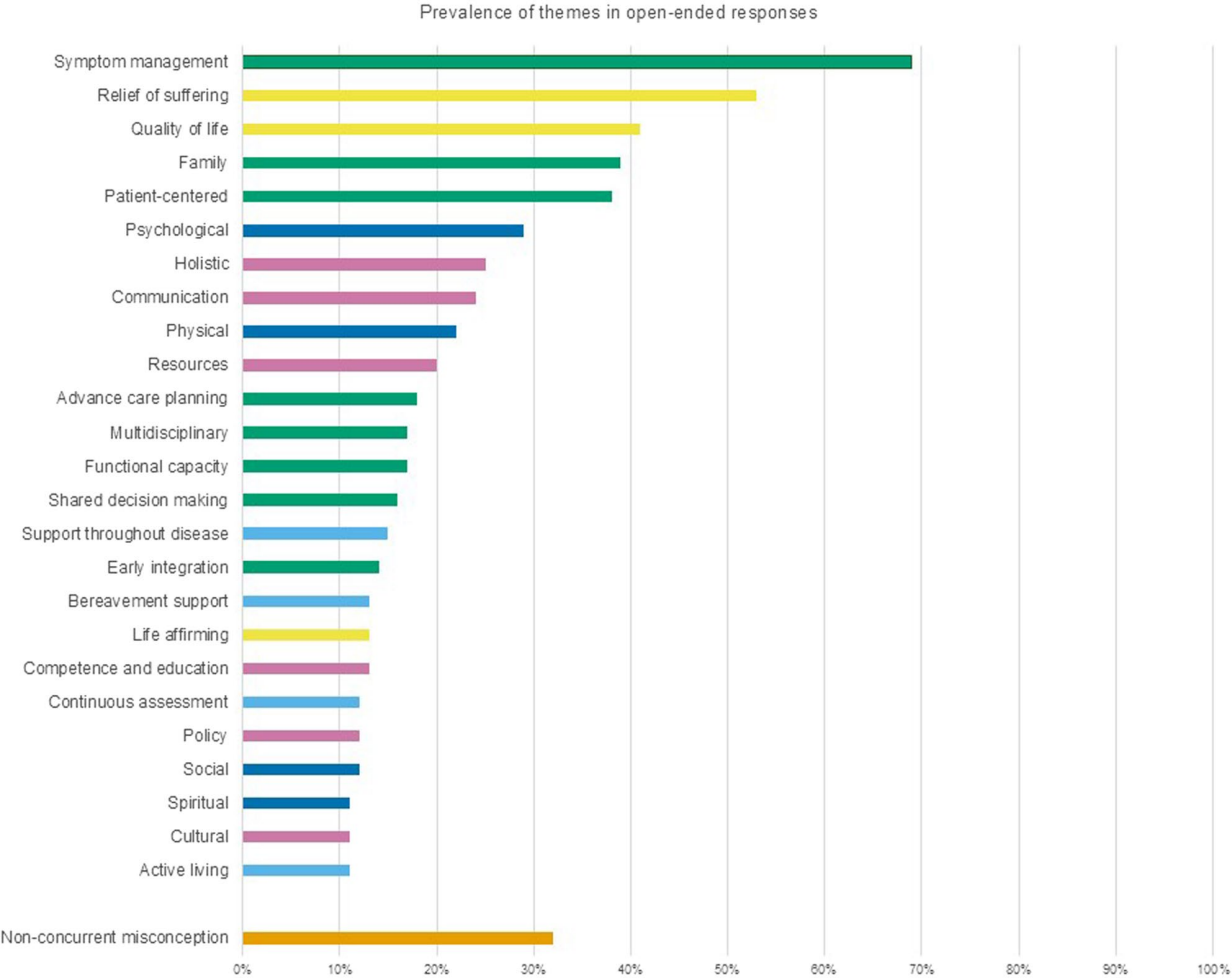
Prevalence of themes in open-ended responses to the question “What is palliative care?”. Responses were deductively binarily coded against 25 framework themes. *N* = 96, including one missing response

For the prevalence of themes, see Table 3; Fig. 2. The theme most often mentioned in open-ended responses was symptom management, mentioned in 69% of responses. Out of all domains, the psychological domain was most often mentioned and prevalent in 29% responses, followed by the physical domain in 22% responses. Of palliative goals, half of the respondents recognized relief of suffering, and 41% described quality of life. Palliative care involved family according to 39% of respondents, and patient-centered care was mentioned by 38%.

**Table 3:** Prevalence of deductively coded framework themes with example statements

No.	Item	*N*	%	Example statement
Domains				
1	Physical	21	22%	*“We assess and care for physical and psychological symptoms holistically.”*
2	Psychological	28	29%	*“Important to give the patient time for emotional and psychological support”*
3	Social	11	12%	*“Focus on good pain management and psychosocial support …”*
4	Spiritual	10	11%	*“Consideration of spiritual needs”*
Goals				
5	Life-affirming	12	13%	“P*alliative care enables focus on cherishing being human*”
6	Quality of life	39	41%	*“… curing the disease is not central*,* but increasing the patient’s quality of life”*
7	Relief of suffering	50	53%	*“… relieving suffering*,* promoting quality of life …”*
Factors				
8	Symptom management	66	69%	*“The importance of symptom care is highlighted in palliative care. “*
9	Early integration	13	14%	*“Palliative care would be good to initiate when the disease is diagnosed”*
10	Functional capacity	16	17%	*“… preventing the loss of ability to walk”*
11	Shared decision making	15	16%	*“Activates the patient to participate in their own care”*
12	Advance care planning	17	18%	*“Anticipatory care plan is important*,* so that everyone is on the same page”*
13	Family support	37	39%	*“Considering family members’ needs and support”*
14	Multidisciplinary	16	17%	*“Multidisciplinary collaboration”*
15	Patient-centered care	36	38%	*“Listen to how the patient wants it”*
System drivers				
16	Competence and education	12	13%	*“Care is holistic and requires engagement and knowledge”*
17	Policy	11	12%	*“It’s a human right and it’s availability and rights should be written in the law”*
18	Communication	23	24%	*“Improving communication can improve co-operation between patients and care personnel”*
19	Resources	19	20%	*“That there’s enough time for discussions and questions”*
20	Holistic view	24	25%	*“For me*,* palliative care is a holistic approach”*
21	Cultural	10	11%	*“Turning focus to quality of life requires cultural change”*
Continuity markers				
22	Continuous needs assessment	11	12%	“A*ssessment of symptom and support needs at every clinical appointment”*
23	Support through illness trajectory	14	15%	*“Continuation of care is highlighted. … It’s clear for the patient who has care responsibility and how to contact them.”*
24	Bereavement support	12	13%	*“Supporting relatives*,* so that they can continue life after saying goodbye”*
25	Active living	10	11%	*“As normal life as possible as an important goal”*
Misconception of palliative care				
26	Non-concurrent	30	32%	*“Symptomatic care when it’s wisest to end active care”*

Several themes were of low prevalence in open-ended responses. Out of “domains”, the least often mentioned were social and spiritual. Of “factors”, only 14% mentioned early integration. Among “continuity markers” and “system drivers”, 12% of responses mentioned policies, and 11% mentioned cultural aspects in palliative care. Of the participants, 13% commented on competence and education in palliative care. Continuous needs assessment, support through the illness trajectory, bereavement support, and support for active living were among the least often mentioned themes, all of which were found in 15% of answers or fewer.

Of the respondents, 32% implied that palliative care is end-of-life care or non-concurrent with active oncological treatment.

### Comparing “misconception” vs. “no misconception” group total score to the open-ended question and survey statements values

**Fig. 3:**
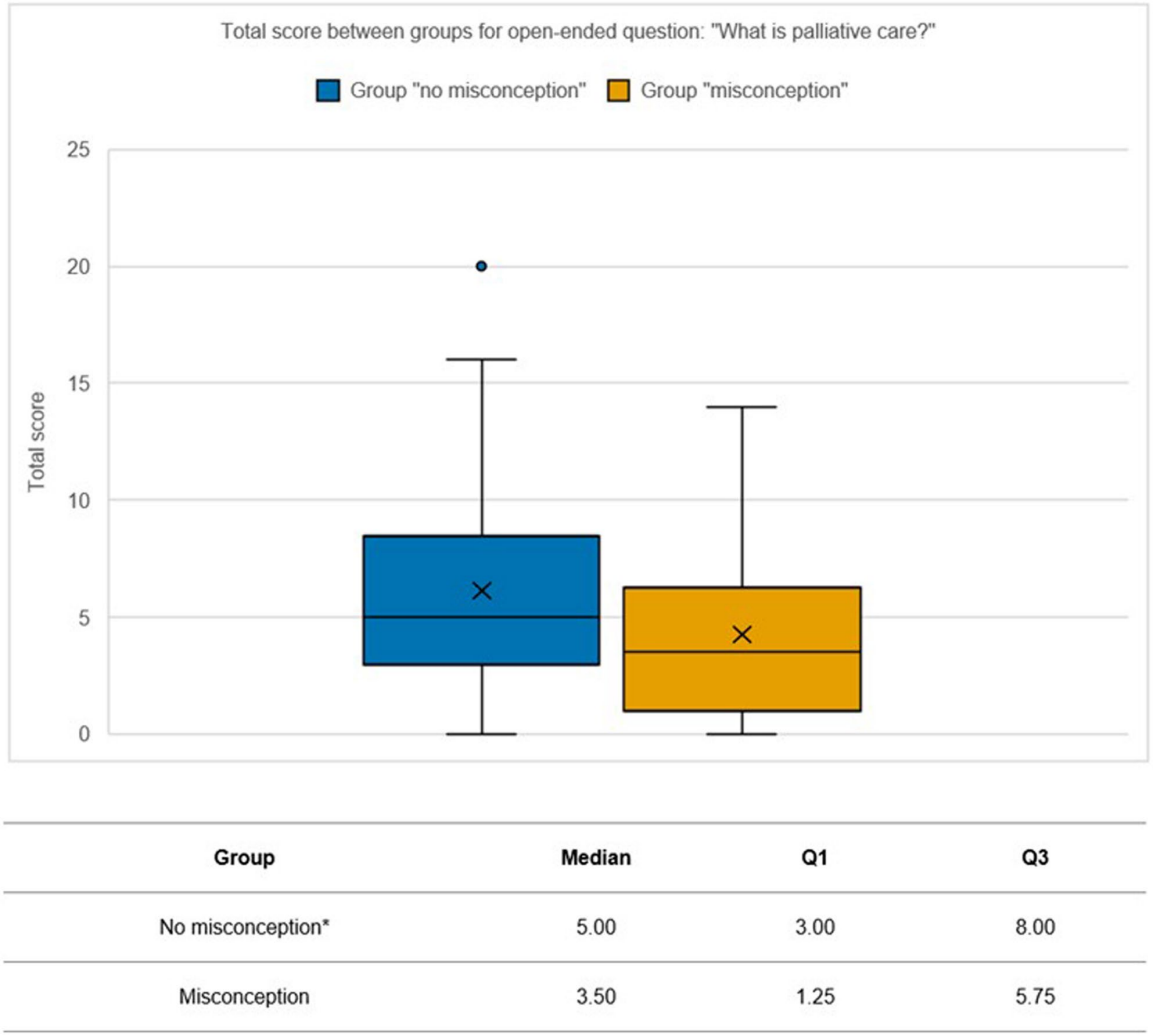
Total score between groups for open-ended question “What is palliative care?”. Total score was calculated by binarily coding responses against 25 framework themes. Participants without the misconception (*n* = 66*) of palliative care as non-concurrent with active oncological treatment had a higher total score on the open-ended question compared to the misconception group (*n* = 30). Mann-Whitney U, *p*-value = 0.028. *Including one missing response. Q1 = Lower quartile. Q3 = Upper quartile

Participants who held the misconception of palliative care as non-concurrent (*n* = 30) with active oncological treatment had significantly lower total scores (*p* = 0.028) in responses to the open-ended question than those who did not (Fig. 3). The “misconception” group had significantly lower total scores for “factors” (*p* = 0.043) and “system drivers” (*p* = 0.009) (Supplementary Fig. 2). No significant differences were observed when analyzing scores for other framework categories and for survey statement values between the “misconception” group and the “no misconception” group. Respondents who conceptualized palliative care as non‑concurrent or terminal‑only differed from those with correct understanding in a limited number of statements. Significant differences were observed in items related to contextual factors (Family support, *p* = 0,039), system‑level drivers (Policy, *p* = 0.017), and continuity of care (Support throughout illness trajectory, *p* = 0.035), see Supplementary Table 2. No significant differences were found in most core conceptual or goal‑related statements, suggesting that misconceptions are not associated with globally lower knowledge but rather with specific structural and continuity‑related aspects of palliative care.

### Triangulation of data

Triangulation of qualitative coding, categorical statement analysis, and Likert‑scale measures suggests that misconceptions about palliative care reflect selective gaps in conceptualizing system‑level and continuity‑related aspects rather than deficits in knowledge, attitudes, or perceived readiness (Table 4). Integration of qualitative prominence, quantitative differences, and effect size patterns demonstrates convergence in core goals and divergence in system-level and practical components. Areas of convergence and divergence between qualitative concept‑coverage in open‑ended responses and quantitative differences by misconception status are presented in Supplementary Table 4. Rank-based effect sizes indicated small-to-moderate differences (*r* = 0.22; δ = 0.28), with an estimated post-hoc power of approximately 52%.

**Table 4:** Alignment between qualitative prominence and quantitative differences across conceptual areas

Conceptual area	Qualitative prominence	Quantitative difference by misconception	Interpretation
Physical and psychological domains	High	No	These domains were consistently emphasized and appear to represent a shared foundational understanding of palliative care
Goals of care (quality of life, relief of suffering)	High	No	Broad agreement across respondents regarding the core aims of palliative care
Family support	Moderate	Yes	Family-centered and relational aspects were less consistently recognized, with variation between groups
Early integration of palliative care	Low	Trend	Palliative care was more often conceptualized in a late or terminal phase rather than as an early, integrated approach
System-level drivers (policy, culture)	Low	Yes	Organizational and structural influences were infrequently addressed, suggesting a system-level blind spot
Continuity across the illness trajectory	Low	Yes	Limited attention to longitudinal involvement of palliative care across the disease course
Knowledge, attitudes, and readiness	High	No	Self-reported competence and readiness were high, despite the presence of conceptual gaps in other areas

## Discussion

In this cross-sectional mixed-methods survey of 96 oncology-service professionals (response rate 88.1%), structured items showed strong endorsement of palliative care benefits and core values, whereas agreement was lower for perceived competence and lowest for perceived resources and support. Free-text definitions revealed substantial variability in conceptual scope, with 32% describing palliative care as end-of-life care and/or as non-concurrent with active oncological treatment. Participants expressing this non-concurrent/end-of-life framing had significantly lower concept-coverage scores in their free-text responses than those without this framing (*p* = 0.028), particularly in categories reflecting practical factors (*p* = 0.043) and system drivers (*p* = 0.009). Notably, endorsement patterns on the Likert items did not differ meaningfully between framing groups, suggesting that the open-ended definition captured conceptual gaps not detected by structured statements alone.

In the present study, palliative care was seen as a valuable part of the cancer patient’s care continuum. Participants emphasized improving QoL and relieving suffering through adequate symptom management. A holistic patient-centered perspective, including the involvement of family and relatives, was recognized as part of palliative care. Overall, healthcare professionals had positive attitudes towards palliative care and saw it as valuable and dignifying. However, agreement was lower on the timing of palliative care initiation and on having enough skills, resources, and support to provide high-quality palliative care. Notably, a substantial portion of participants perceived palliative care as non-concurrent with active oncological treatment or equal to end-of-life care, and the healthcare professionals who held this perception had lower knowledge of aspects influencing palliative care.

There was a discrepancy found in the results regarding when palliative care should be provided. Although healthcare personnel, according to survey statements, perceived that early initiation improves QoL and disagreed that palliative care should be initiated in the last weeks of life, almost one-third of open-ended responses showed a contrasting perception. Wallerstedt et al. conducted focus group interviews with 74 healthcare professionals in Sweden, showing similar confusion [17]. The participants in the Wallerstedt study had an unclear understanding of what palliative care indicates, using end-of-life terminology while still recognizing that the palliative care phase can stretch from days up to years. McMillan et al. investigated 73 physicians’ knowledge and attitudes toward palliative care in South Africa, where 93% recognized that palliative care should be concurrent with active oncological treatment, but only half of the participants introduced palliative care early [22]. Magalhães et al. conducted a systematic review of healthcare professionals’ perception of palliative care around the world in 2024, in which the Wallerstedt study was included [21]. However, they didn’t compare perceptions and knowledge of palliative care according to the WHO definition. Further limitations to the systematic review include not providing the theoretical lens through which the review was conducted. Our approach ensured that early integration, advance care planning, shared decision-making, competence, and resource considerations were systematically addressed, offering practical implications for policy and clinical practice [4]. Inclusion of THL’s recommendations allowed for a more nuanced interpretation than WHO’s definition alone, which has been criticized for being outdated and insufficiently reflective of evolving care models [25]. There is also ongoing debate about the adequacy of current definitions, such as International Association for Hospice and Palliative Care (IAHPC) call to update WHO’s definition and European Society for Medical Oncology (ESMO) proposing the usage of a new concept “patient-centered care”, based on both WHO and Multinational Association of Supportive Care in Cancer (MASCC) definitions [2, 25]. This suggests that existing frameworks may have conceptual gaps, which could limit the completeness of our analysis. The Finnish national quality recommendations published in 2022 by Saarto et al. conclude that the integrated model of early concurrent palliative care is yet to be fully implemented in Finland [26]. This could partly explain why the participants in our study still held the misconception of palliative care as non-concurrent with active oncological treatment.

Open-ended responses, together with endorsement patterns in survey statements, implied that some favorable views may have reflected an end-of-life-focused interpretation of palliative care, rather than full alignment with the WHO definition. Healthcare personnel’s attitudes towards palliative care and end-of-life care have generally been positive, even when levels of knowledge were low. Kassa et al. investigated nurses’ attitudes and knowledge towards palliative care in Ethiopia, measured with Palliative Care Quiz of Nursing (PCQN) and Frommelt Attitude Toward Care of the Dying (FATCOD) scales, where 3/4 of nurses had a favorable attitude towards palliative care, although knowledge was low as assessed by PCQN [27]. In a systematic review of 13 articles on nurses’ attitudes towards death and dying by Jeong et al., the findings implied, however, that higher knowledge indicated more positive attitudes [28]. Adolfsson et al. surveyed Swedish physicians’ attitudes, practices, and experiences towards early palliative care [18]. The physicians had positive attitudes toward early palliative care, although remarkably, this did not reflect in their behavior, as referrals to palliative care were still made late. Wendlandt et al. showed similar results, where physicians in Canada had positive attitudes toward early integration of palliative care, but still referred at a late stage [29]. This implies that positive attitudes toward palliative care do not automatically reflect favorable behavior, i.e., early integration, nor a comprehensive understanding of the concept as defined by the WHO [1].

A novel perspective in our study was investigating self-perceived readiness among healthcare personnel to provide palliative care. Readiness statements for having enough resources, emotional support, and skills had the lowest mean scores in the survey. Similar findings were reported by Sorensen et al. among Canadian physicians, where only half of the participants agreed to having enough resources, and almost a third disagreed with the statement [30]. Multivariate analysis showed higher agreement among the physicians working in teams. Multiple service options available for patient referral, including communal services, were also associated with a higher agreement to the statement. Palliative care physicians in a study by Sue-A-Quan et al., however, felt that most palliative care needs could primarily be provided within primary palliative care, but that resources in primary care were deficient [31]. Discussing perceived skills and competence, nursing students in Finland perceived their competence as least sufficient in multicultural and existential aspects out of the provided elements [32], which could reflect why similar themes in the open-ended responses in our study were of low prevalence. These findings underscore the need for structural resource assessment and competence development to ensure readiness to provide care in all aspects of palliative care.

Participants in the group without the misconception had higher concept coverage scores in the open-ended question, both overall and among framework themes within “factors” and “system drivers”. Practical factors such as shared decision-making, advance care planning, and a multidisciplinary approach were thus not recognized to the same extent as symptom management, although all are valuable aspects of palliative care [2, 26]. This pattern suggests that the open-ended question captured dimensions of conceptualizations that were not well differentiated by the structural statements. However, the low total scores among both groups still show the need for further education on the scope of palliative care in Finland, as is also concluded by Saarto et al. [26].

### Strengths and limitations

The mixed methodology of this study provides a nuanced understanding of knowledge, attitudes, and readiness among healthcare professionals. Compared to a previous study on healthcare personnel’s perceptions of palliative care, our study systematically compared palliative care understanding in relation to the WHO definition of palliative care [17]. A key strength of our study is the large sample of responses to the open-ended question “What is palliative care?”.

Another key strength of this study is the use of a framework to guide qualitative analysis. We based the deductive coding process on WHO’s definition of palliative care, in addition to elements from the mentioned Finnish national quality recommendations, which provide criteria for culturally sensitive, patient-centered care and advance care planning in the Finnish context [26]. This combination ensured that the analysis was grounded in internationally recognized principles while remaining relevant to local practice.

We recognize that the instrument used in this study has yet to be validated. In addition, only 9 males participated in the study. Thus, the statistically significant results between genders weren’t discussed, as they might not fully represent all male healthcare professionals’ perspectives on palliative care in Ostrobothnia. The low prevalence of males participating in our study, however, reflects a gender imbalance in the Ostrobothnian workforce in oncology and palliative care. In addition, the heterogeneous group of healthcare professionals implies varying baseline exposure to palliative care, whilst simultaneously reflecting real-world healthcare. Generalisability is limited to a single region, and sample heterogeneity reflects professional roles within the same setting.

Furthermore, our recruitment context may have attracted staff with greater baseline interest in palliative care and therefore lead to underestimation of misconception prevalence among healthcare professionals.

The low prevalence of some themes in open-ended answers could be addressed. Although the format of the survey was planned thoroughly, including only one open-ended question, survey fatigue and lack of motivation could still have contributed to incomplete answers in the open-ended question [33, 34]. Binary concept coverage is sensitive to response length. We therefore conducted sensitivity analyses adjusting for response length and repeating comparisons in a length-restricted subsample. Conclusions were directionally consistent. Given the exploratory design, the sample size was seen as sufficient for descriptive and non-parametric analyses, but not powered to detect small effect sizes.

The authors also acknowledge that their clinical backgrounds in palliative care may have influenced data interpretation; however, data collection and recruitment was conducted by a medical student and an early-career resident, and reflexivity was addressed through team-based analysis and ongoing critical discussion.

### Research implications

For a deeper understanding of healthcare personnel’s knowledge, focus group interviews could broaden the understanding of healthcare personnel’s perception of palliative care compared to concepts described in guidelines and definitions. This study indicates a measurement problem that should be treated as a research target, not only an educational target. Open-ended items differentiated conceptual scope and timing, while structural endorsement items showed ceiling effects for several normative and benefit statements.

## Conclusion

Our findings suggest that there is a misconception among healthcare personnel that palliative care is end-of-life care or non-concurrent with active oncological treatment in Finland. Attitudes among healthcare professionals towards palliative care are positive, but there is a need for definition- and guideline-based educational interventions to Finnish healthcare personnel on palliative care to enable early integration, as the WHO and guidelines recommend.

## Supplementary Information

Supplementary Material 1.

## Data Availability

The datasets generated and/or analyzed during the current study are not publicly available because they consist of potentially identifiable free-text responses from a small regional workforce and are therefore subject to GDPR-related privacy considerations. De-identified data are available from the corresponding author on reasonable request, subject to institutional data access procedures and approval.

## Abbreviations

ACP: Advance care planning
FATCOD: Frommelt Attitude Toward Care of the Dying
IQR: Interquartile Range
PCQN: Palliative Care Quiz of Nursing
QoL: Quality of life
SDM: Shared decision making
THL: Finnish Institute for Health and Welfare
WHO: World Health Organization
HS: HP–Hopea–Silver Healthcare Professional
