# Supplementary material for: Healthcare professionals’ conceptualizations of palliative care and readiness for early integration: a cross‑sectional mixed‑methods survey in Finland
Source: BMC Palliat Care. 2026 Jun 16;25:179. doi: 10.1186/s12904-026-02194-x (PMC13283322; doi:10.1186/s12904-026-02194-x)

**Supplementary material**

**Manuscript title: *Healthcare professionals’ conceptualizations of palliative care and readiness for early integration: a cross‑sectional mixed‑methods survey in Finland***

***Authors:*** Ebba Åström^1,2*^, Ella Saaranen^2,3*^, Heidi Andersén^2,3^, Mikael Johansson^1^, Nelli Nåhls^2^

^1^ Department of Diagnostics and Intervention, Oncology, Umeå University, Umeå, Sweden

^2^ Vaasa Oncology Clinic, Wellbeing Services of Ostrobothnia, Vaasa, Finland

^3^ Department of Clinical Oncology, Turku University, Turku, Finland

*Shared first authorship Ebba Åström and Ella Saaranen


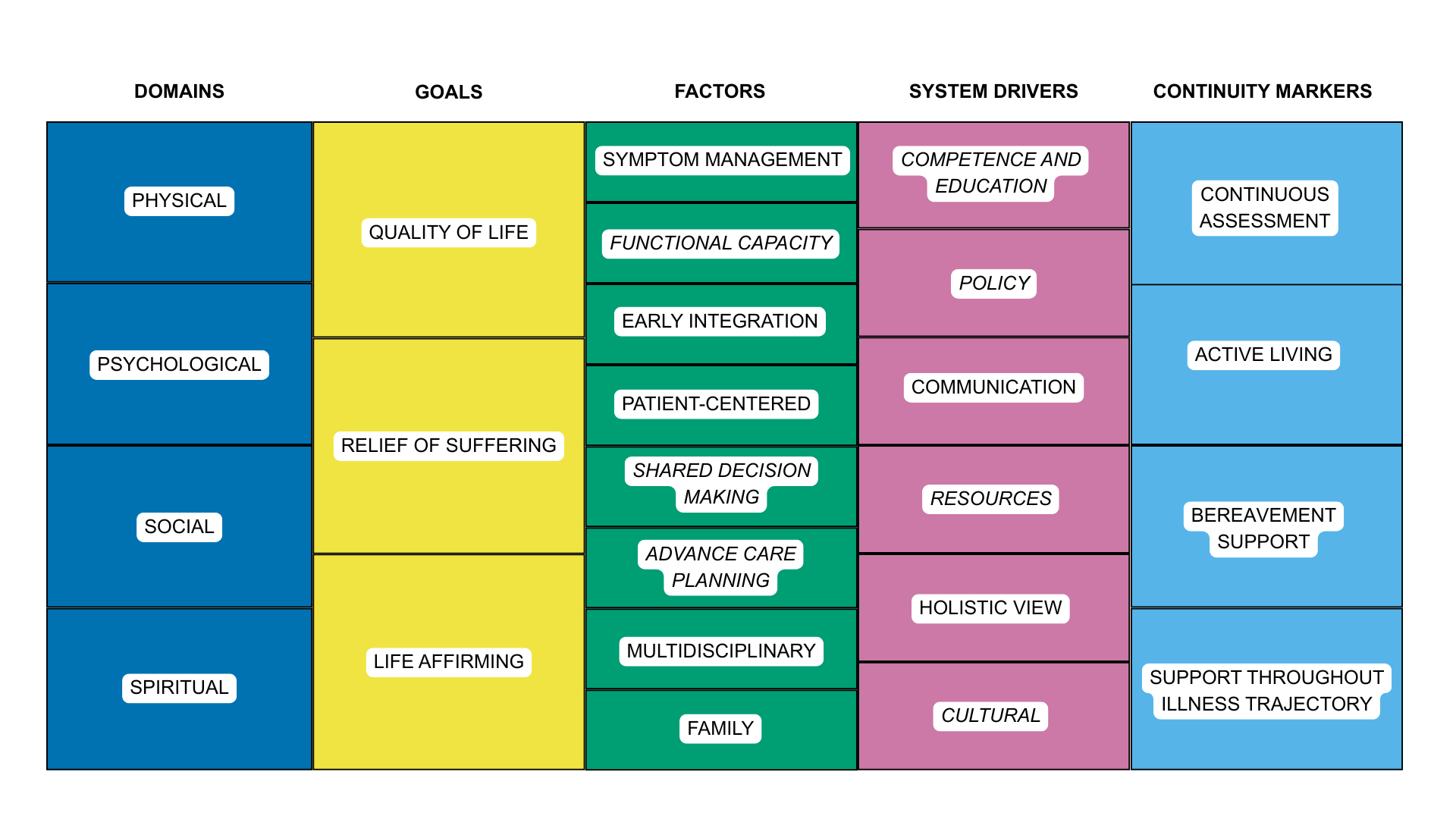


**Supplementary figure 1. Conceptual framework for binary deductive analysis of the open-ended question “What is palliative care?"**.

Participants received 1 point per theme mentioned in open-ended responses, yielding a total score of 25 possible and 3-8 per category, depending on how many themes there are in each category. Categories are divided by color. Themes written in normal font are derived from the WHO definition of palliative care (1). Themes written in italics are derived from Finnish National Quality Recommendations as described by Saarto *et al*. (25).


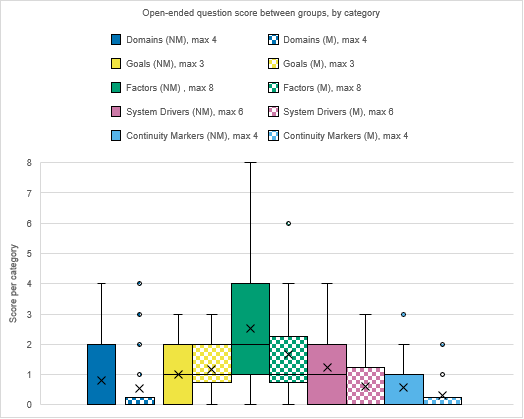


**Supplementary figure 2. Scores in participants’ responses to the open-ended question “What is palliative care?”, divided by category.** Scores for each category were higher for participants in the group without the misconception (NM) of palliative care as non-concurrent with active oncological treatment. Statistically significant for “Factors” (p = 0.043) and “System Drivers” (p = 0.009), Mann-Whitney U. M = misconception group (n =30).

| Statement | Males  (N = 9) | Females  (N = 87) | P value* | Finnish speaking (n = 53) | Swedish speaking (n = 43) | P value* | Lower education (n = 66) | Higher education  (n = 30) | P value* | Lower age group (ages 18-39)  (n = 34) | Higher age group (ages 40-60+)  (n = 62) | P value* |
| --- | --- | --- | --- | --- | --- | --- | --- | --- | --- | --- | --- | --- |
| Mean (SD) | Mean (SD) | Mean (SD) |  | Mean (SD) | Mean (SD) |  | Mean (SD) | Mean (SD) |  | Mean (SD) | Mean (SD) |  |
| Concurrent | 3.3 (1.4) | 3.7 (1.3) | 0.363 | 3.9 (1.2) | 3.5 (1.4) | 0.159 | 3.6 (1.4) | 4.0 (1.1) | 0.275 | 3.6 (1.3) | 3.8 (1.3) | 0.359 |
| Early QoL | 4.8 (0.4) | 4.8 (0.5) | 0.381 | 4.9 (0.4) | 4.7 (0.7) | 0.110 | 4.8 (0.6) | 4.9 (0.3) | 0.236 | 4.8 (0.4) | 4.8 (0.6) | 0.298 |
| Drug dependency | 4.8 (0.4) | 4.6 (0.7) | 0.611 | 4.8 (0.6) | 4.5 (0.8) | 0.050 | 4.5 (0.8) | 4.8 (0.5) | **0.031*** | 4.5 (0.7) | 4.7 (0.7) | 0.235 |
| Prolong life | 4.4 (0.9) | 4.4 (0.8) | 0.846 | 4.5 (0.8) | 4.4 (0.8) | 0.468 | 4.4 (0.8) | 4.5 (0.9) | 0.329 | 4.3 (0.9) | 4.5 (0.8) | 0.235 |
| Psychosocial spiritual | 4.7 (0.5) | 4.9 (0.4) | 0.134 | 4.9 (0.4) | 4.8 (0.5) | 0.838 | 4.8 (0.4) | 4.8 (0.5) | 0.730 | 4.8 (0.5) | 4.9 (0.3) | 0.301 |
| Late initiation | 5.0 (0.0) | 4.8 (0.5) | 0.286 | 4.9 (0.6) | 4.8 (0.4) | 0.320 | 4.9 (0.3) | 4.8 (0.8) | 0.492 | 4.8 (0.5) | 4.9 (0.5) | 0.092 |
| Death preparation | 4.0 (1.0) | 4.6 (0.6) | 0.062 | 4.6 (0.6) | 4.4 (0.8) | 0.409 | 4.5 (0.7) | 4.5 (0.7) | 0.673 | 4.4 (0.8) | 4.6 (0.6) | 0.157 |
| Family participation | 4.4 (0.7) | 4.8 (0.4) | 0.085 | 4.8 (0.5) | 4.7 (0.5) | 0.303 | 4.8 (0.5) | 4.7 (0.5) | 0.914 | 4.7 (0.5) | 4.8 (0.5) | 0.891 |
| Communication | 4.6 (0.7) | 4.9 (0.3) | 0.103 | 4.8 (0.4) | 4.9 (0.4) | 0.696 | 4.9 (0.4) | 4.8 (0.5) | 0.825 | 4.8 (0.4) | 4.8 (0.4) | 0.740 |
| Improve QoL | 4.9 (0.3) | 4.9 (0.3) | 0.852 | 4.9 (0.3) | 4.9 (0.3) | 0.497 | 4.9 (0.3) | 4.9 (0.3) | 0.888 | 4.9 (0.4) | 4.9 (0.2) | 0.187 |
| Equal domains | 3.9 (0.6) | 4.7 (0.5) | **< 0.001*** | 4.7 (0.6) | 4.6 (0.6) | 0.574 | 4.7 (0.6) | 4.6 (0.6) | 0.351 | 4.6 (0.6) | 4.7 (0.6) | 0.454 |
| Collaboration | 4.6 (0.5) | 4.9 (0.4) | **0.009*** | 4.8 (0.4) | 4.8 (0.5) | 0.496 | 4.9 (0.5) | 4.8 (0.4) | 0.333 | 4.9 (0.4) | 4.8 (0.5) | 0.995 |
| Skills | 3.1 (1.0) | 3.9 (0.9) | **0.020*** | 3.8 (0.9) | 3.9 (1.0) | 0.692 | 3.8 (0.9) | 4.0 (1.0) | 0.248 | 3.7 (1.0) | 3.9 (1.0) | 0.327 |
| Resources | 2.9 (0.9) | 3.5 (0.9) | **0.033*** | 3.4 (0.8) | 3.5 (1.0) | 0.522 | 3.4 (0.9) | 3.7 (1.0) | 0.145 | 3.2 (0.9) | 3.6 (0.9) | **0.045*** |
| Emotional | 3.1 (0.9) | 3.8 (0.9) | 0.038* | 3.8 (0.9) | 3.6 (1.0) | 0.157 | 3.6 (0.9) | 3.9 (0.9) | 0.113 | 3.5 (0.9) | 3.8 (1.0) | 0.135 |

**Supplementary table 1. Likert-scale comparison between genders, languages, educational level, and age groups**

*Kruskal-Wallis’ test

QoL = Quality of Life

**Supplementary table 2. Understanding of palliative care statements by misconception status (N = 96)**

| No. | Item | N (%) | No misconception n = 66 n (%) | Misconception n = 30 n (%) | p value |
| --- | --- | --- | --- | --- | --- |
| 1 | Physical | 21 (22) | 17 (25.8) | 4 (13.3) | 0.172 |
| 2 | Psychological | 28 (29) | 21 (31.8) | 7 (23.3) | 0.397 |
| 3 | Social | 11 (12) | 7 (10.6) | 4 (13.3) | 0.697 |
| 4 | Spiritual | 10 (11) | 9 (13.6) | 1 (3.3) | 0.126 |
| 5 | Life-affirming | 12 (13) | 6 (9.1) | 6 (20.0) | 0.134 |
| 6 | Quality of life | 39 (41) | 27 (40.9) | 12 (40.0) | 0.933 |
| 7 | Relief of suffering | 50 (53) | 33 (50.0) | 17 (56.7) | 0.544 |
| 8 | Symptom management | 66 (69) | 46 (69.7) | 20 (66.7) | 0.767 |
| 9 | Early integration | 13 (14) | 12 (18.2) | 1 (3.3) | 0.049 |
| 10 | Functional capacity | 16 (17) | 14 (21.2) | 2 (6.7) | 0.076 |
| 11 | Shared decision making | 15 (16) | 11 (16.7) | 4 (13.3) | 0.677 |
| 12 | Advance care planning | 17 (18) | 13 (19.7) | 4 (13.3) | 0.449 |
| 13 | Family support | 37 (39) | 30 (45.5) | 7 (23.3) | **0.039** |
| 14 | Multidisciplinary | 16 (17) | 13 (19.7) | 3 (10.0) | 0.237 |
| 15 | Patient-centered care | 36 (38) | 27 (40.9) | 9 (30.0) | 0.306 |
| 16 | Competence and education | 12 (13) | 9 (13.6) | 3 (10.0) | 0.618 |
| 17 | Policy | 11 (12) | 11 (16.7) | 0 (0.0) | **0.017** |
| 18 | Communication | 23 (24) | 18 (27.3) | 5 (16.7) | 0.259 |
| 19 | Resources | 19 (20) | 16 (24.2) | 3 (10.0) | 0.105 |
| 20 | Holistic view | 24 (25) | 18 (27.3) | 6 (20.0) | 0.446 |
| 21 | Cultural | 10 (11) | 9 (13.6) | 1 (3.3) | 0.126 |
| 22 | Continuous needs assessment | 11 (12) | 10 (15.2) | 1 (3.3) | 0.092 |
| 23 | Support throughout illness trajectory | 14 (15) | 13 (19.7) | 1 (3.3) | **0.035** |
| 24 | Bereavement support | 12 (13) | 7 (10.6) | 5 (16.7) | 0.405 |
| 25 | Active living | 10 (11) | 8 (12.1) | 2 (6.7) | 0.417 |

**Supplementary table 3. Reorganization of the predefined deductive framework into higher-order thematic domains for presentation purposes.**

| Higher-order thematic domain | Original framework categories | Included themes |
| --- | --- | --- |
| Core meaning of palliative care | Domains | Physical, psychological, social, and spiritual domains |
| Goals of palliative care | Goals | Relief of suffering; quality of life; life affirming orientation |
| Scope and timing across the illness trajectory | Continuity markers: Factors (early integration) | Early integration; continuity of care; support throughout the illness trajectory; active living; continuous needs assessment; bereavement support |
| Practical enactment in clinical care | Factors | Symptom management; functional capacity; shared decision-making; advance care planning; family support; multidisciplinary collaboration; patient-centered care |
| System-level and contextual conditions | System drivers | Competence and education; policy; resources; communication; cultural factors |

**Supplementary Table S4. Joint display integrating qualitative theme prominence, quantitative differences, and interpretive synthesis**

| Conceptual area | Qualitative prominence in open‑ended responses | Quantitative difference by misconception status | Effect size (r / Cliff’s δ) | Integrated interpretation |
| --- | --- | --- | --- | --- |
| Core meaning of palliative care | High prominence; physical and psychological domains consistently articulated; social and spiritual domains less frequent | No significant difference | r ≈ 0.11; δ ≈ 0.12 | Fundamental understanding of palliative care domains is broadly shared regardless of misconception status |
| Goals of palliative care | High prominence; relief of suffering and quality of life frequently mentioned | No significant difference | r ≈ 0.10; δ ≈ 0.12 | Shared endorsement of core goals suggests misconceptions do not reflect disagreement about aims of care |
| Timing and scope across the illness trajectory | Low prominence; early integration, continuity, and longitudinal support infrequently articulated | Trends but no consistent statistical separation | r ≈ 0.14; δ ≈ 0.15 | Longitudinal and trajectory‑based aspects of palliative care are weakly conceptualized overall |
| Practical enactment in clinical care | Moderate prominence; symptom management common, while proactive elements (e.g. ACP, shared decision‑making) less frequent | Significant difference | **r = 0.21; δ = 0.25** | Misconceptions are associated with reduced recognition of proactive and structured care processes |
| System‑level and contextual conditions | Lowest prominence of all domains | Strongest significant difference | **r = 0.27; δ = 0.32** |  |

Effect sizes are reported as rank‑based *r* and Cliff’s delta (δ). Values around 0.10 are considered small, 0.30 moderate, and 0.50 large. Quantitative findings are interpreted in conjunction with qualitative prominence to support mixed‑methods triangulation.


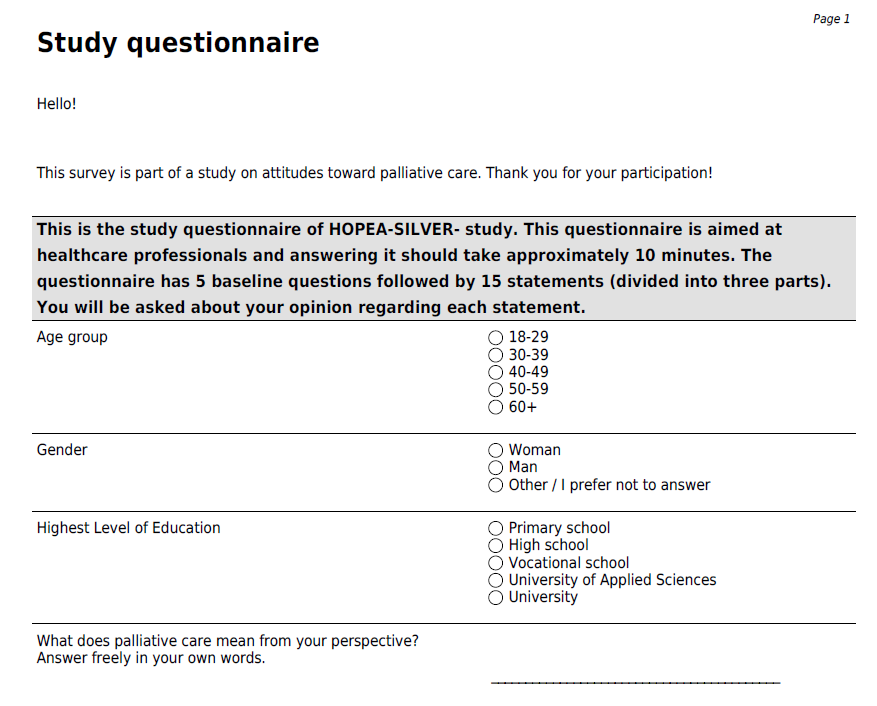


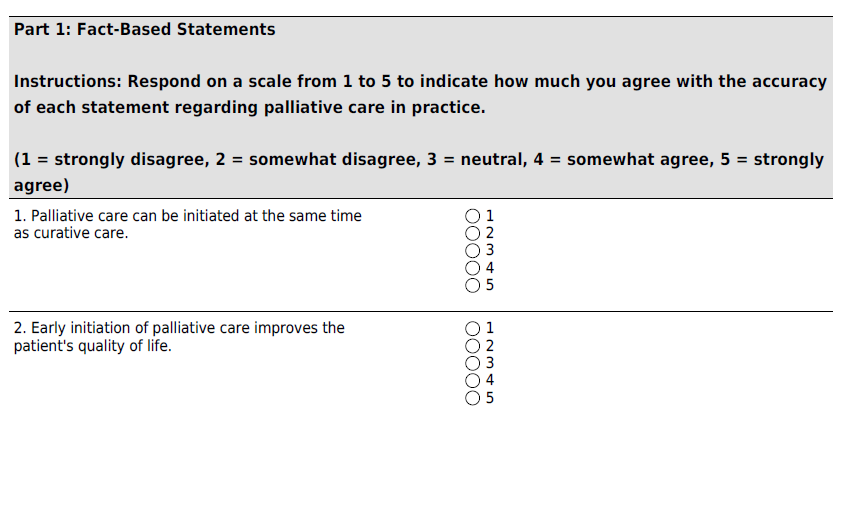


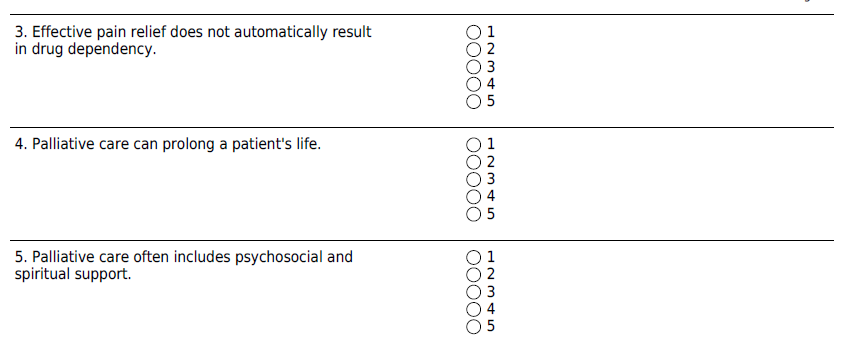


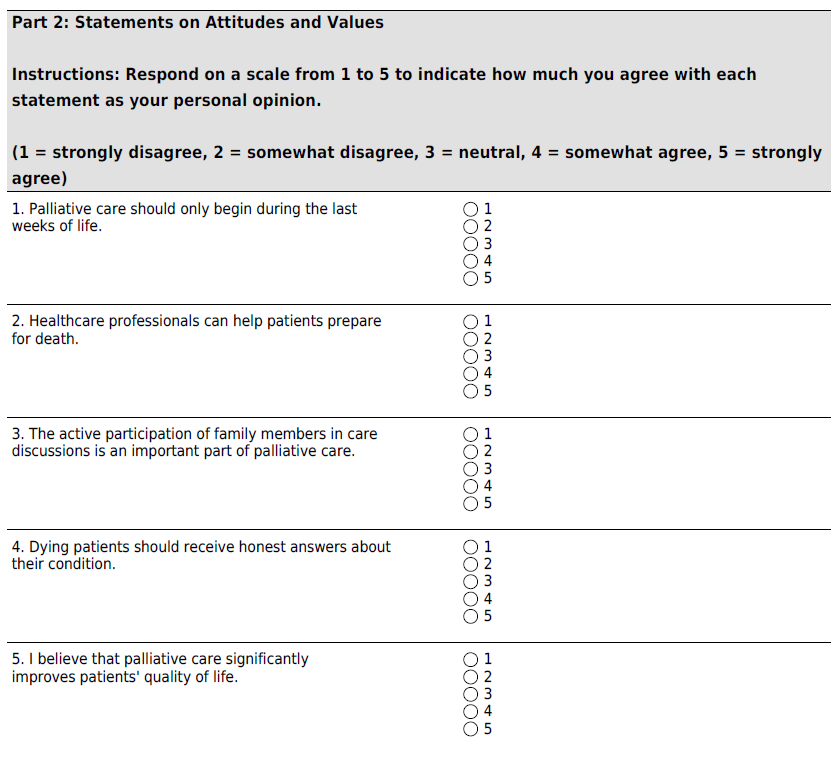


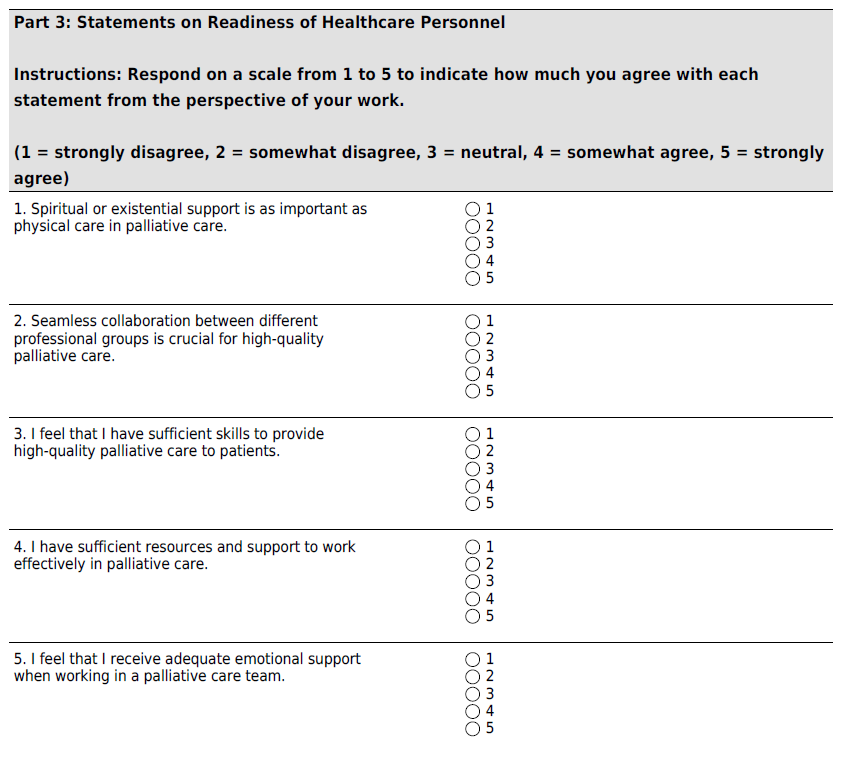


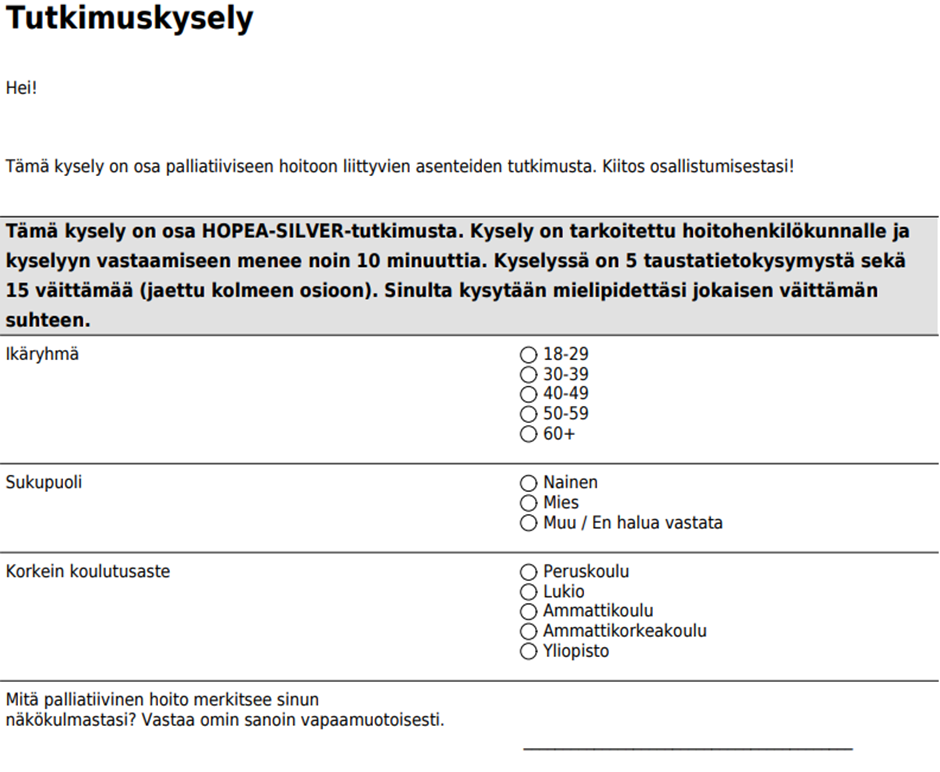


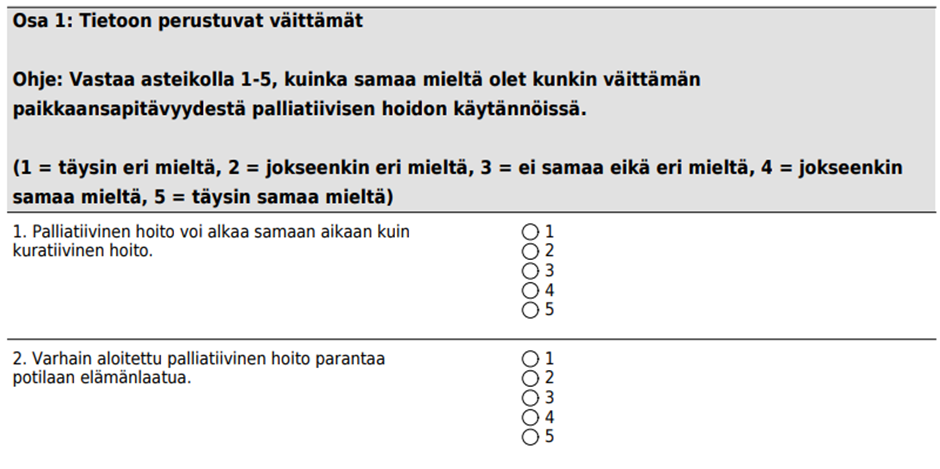


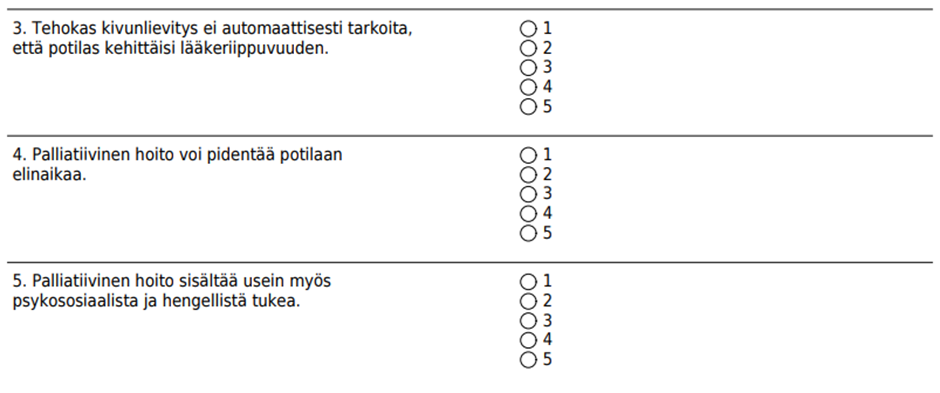


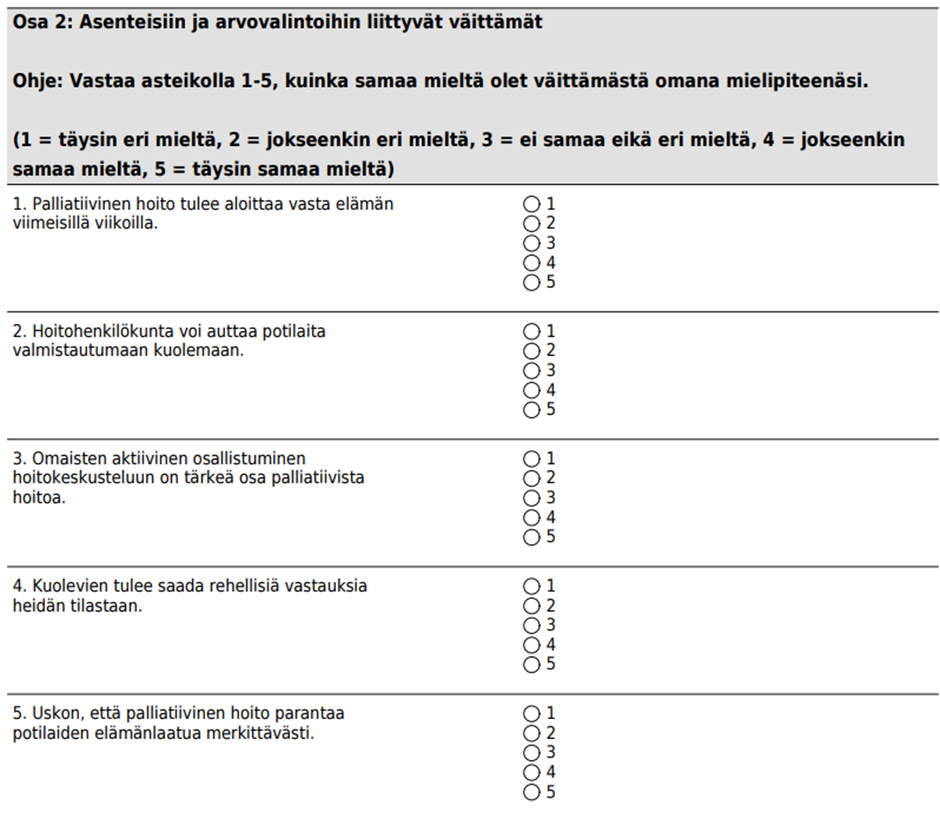


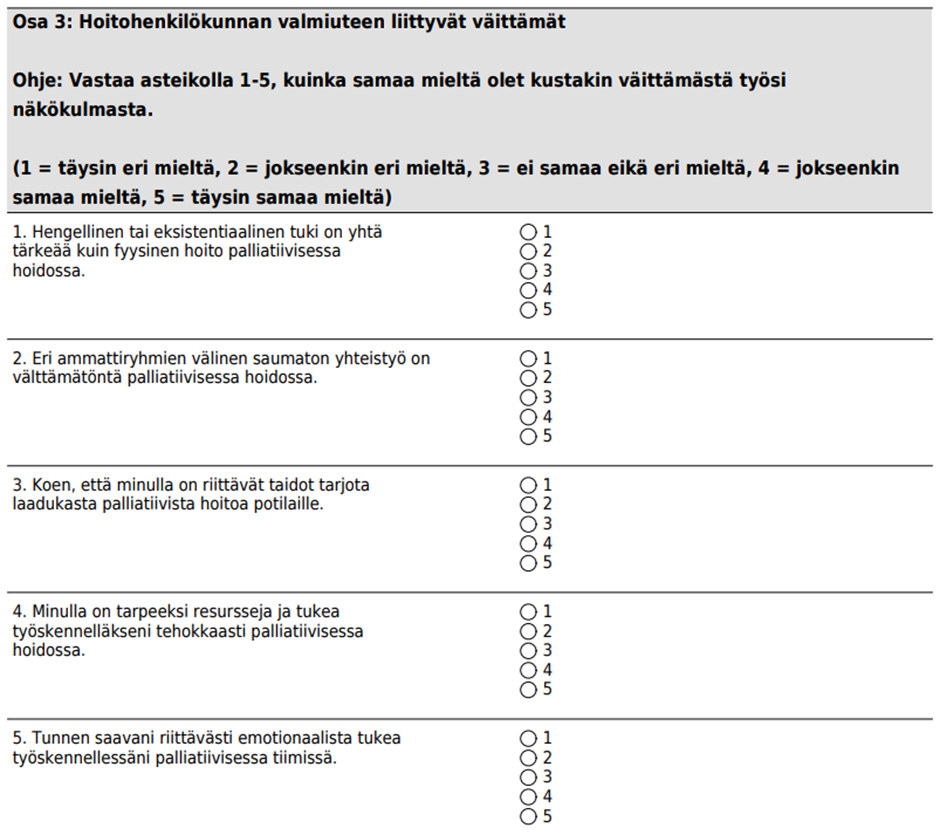


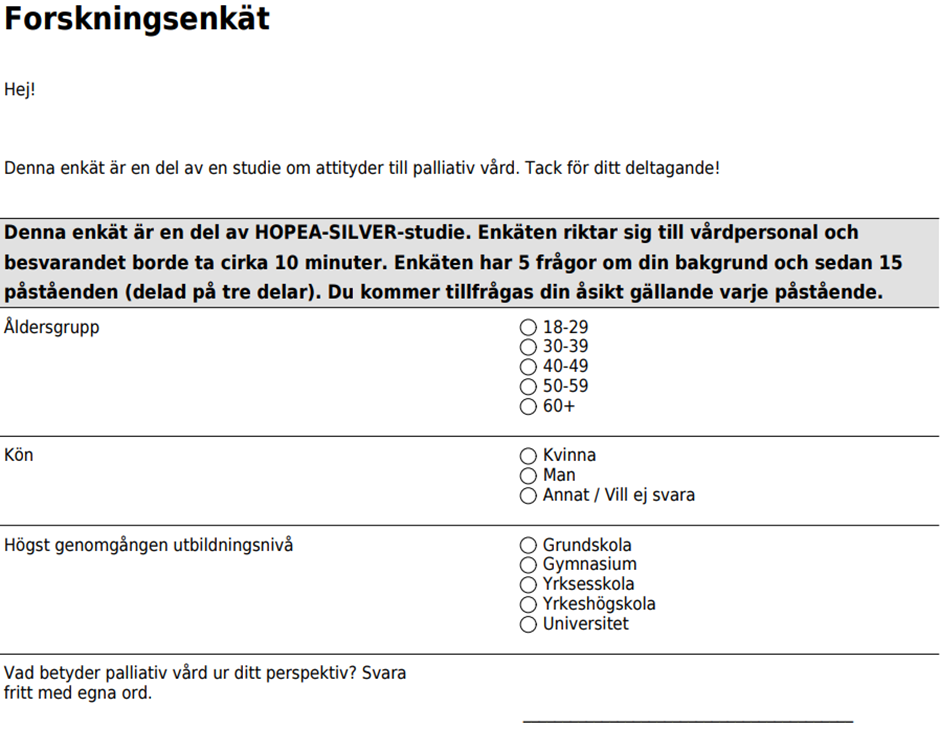

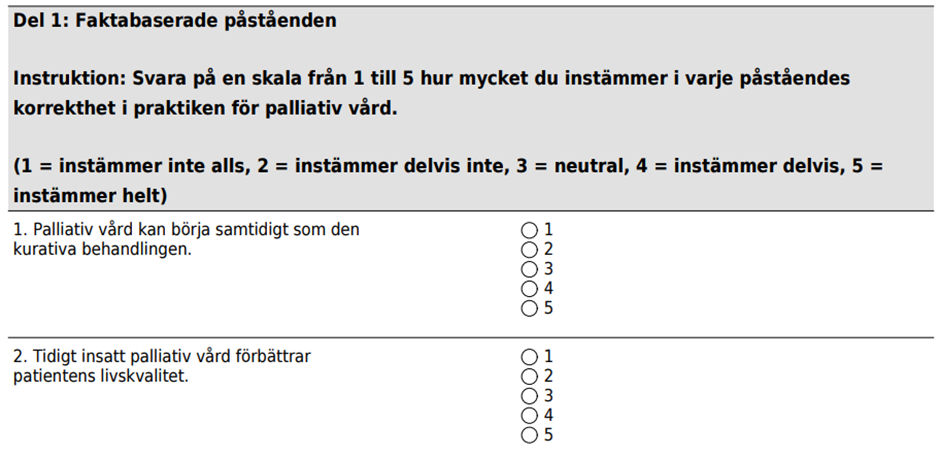


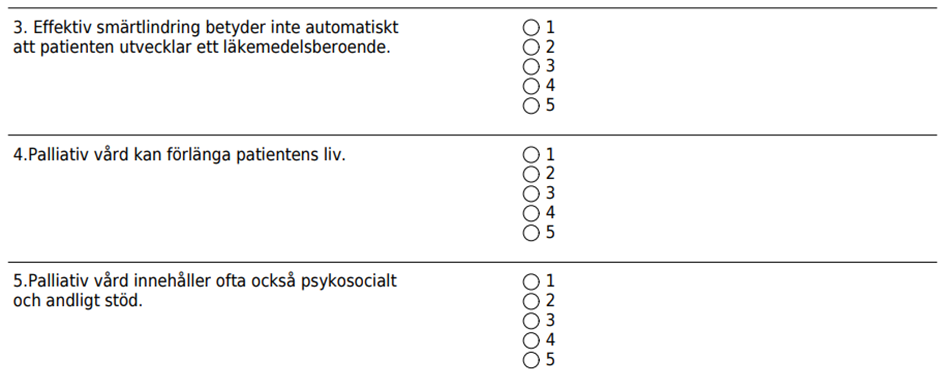

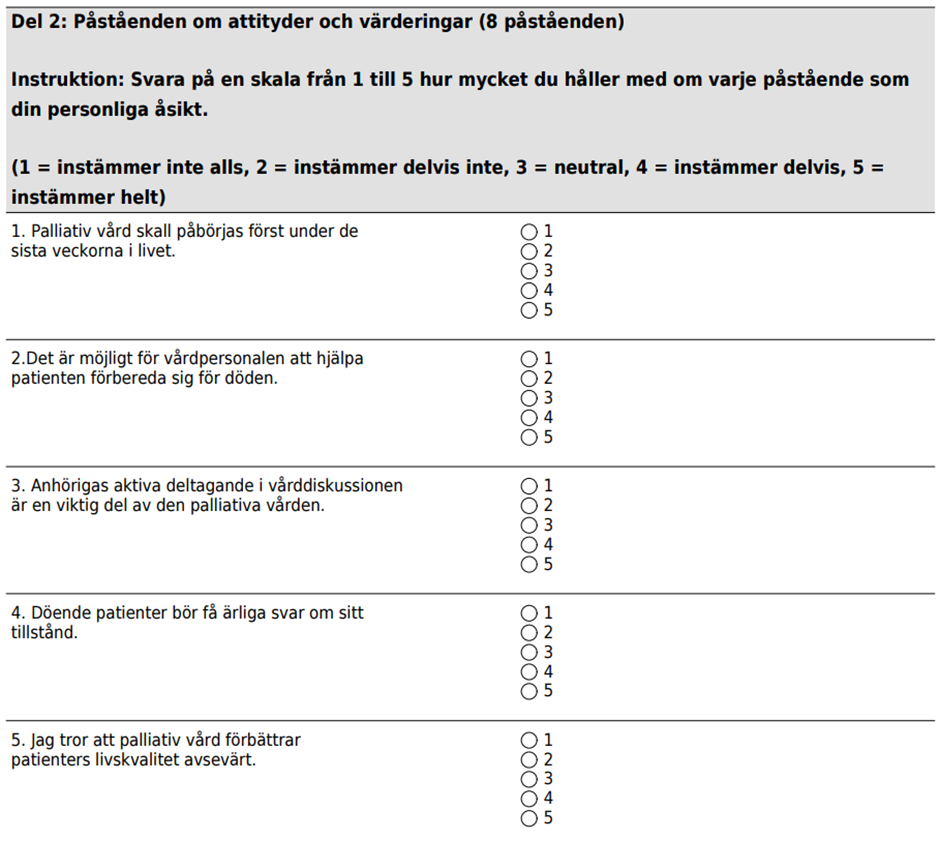

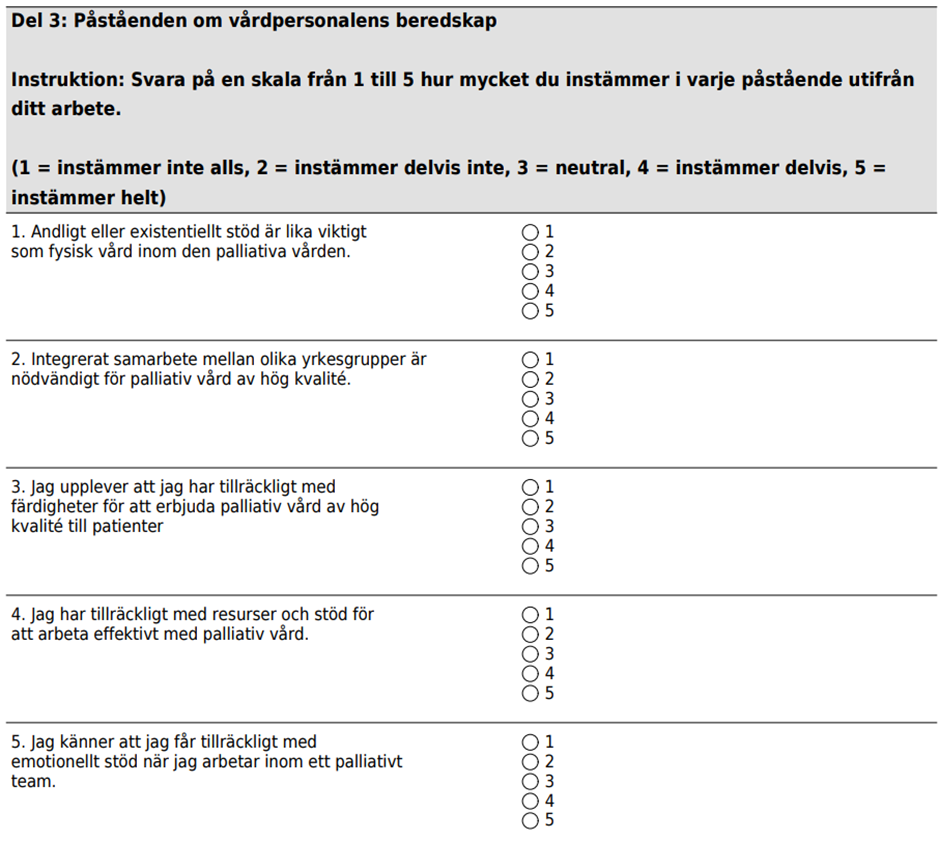

Supplement: Supplementary file 1 — Supplementary Material 1. [file 12904_2026_2194_MOESM1_ESM.docx]
